# Learning With a Double-Edged Sword? Beneficial and Detrimental Effects of Learning Tests—Taking a First Look at Linkages Among Tests, Later Learning Outcomes, Stress Perceptions, and Intelligence

**DOI:** 10.3389/fpsyg.2021.693585

**Published:** 2021-08-31

**Authors:** Kristin Wenzel, Marc-André Reinhard

**Affiliations:** Department of Psychology, University of Kassel, Kassel, Germany

**Keywords:** learning tests, desirable difficulties, acute stress perceptions, intelligence, long-term learning

## Abstract

It has often been shown that tests as intentionally hindered and difficult learning tasks increase long-term learning compared to easier tasks. Previous work additionally indicated that higher intelligence might serve as a prerequisite for such beneficial effects of tests. Nevertheless, despite their long-term learning effects, tests were also found to be evaluated as more negative and to lead to more stress and anxiety compared to easier control tasks. Stress and anxiety, in turn, often yield detrimental effects on learning outcomes. Hence, we hypothesized that tests increase later learning outcomes but simultaneously also lead to more stress perceptions. Such increased stress was, in turn, hypothesized to reduce later learning outcomes (thus, stress might serve as a mediator of the beneficial effects of tests on learning). All these assumed effects should further be moderated by intelligence, insofar as that higher intelligence should increase beneficial effects of tests on learning, should decrease stress perceptions caused by tests, and should reduce detrimental effects of stress on learning outcomes. Higher intelligence was also assumed to be generally associated with higher learning. We conducted a laboratory study (*N*=89) to test these hypotheses: Participants underwent an intelligence screening, then worked on either a test or a re-reading control task, and reported their immediate stress perceptions. Later learning outcomes were assessed after 1week. The results supported all assumed main effects but none of the assumed interactions. Thus, participants using tests had higher long-term learning outcomes compared to participants using re-reading tasks. However, participants using tests also perceived more immediate stress compared to participants that only re-read the materials. These stress perceptions in turn diminished the beneficial effects of tests. Stress was also generally related to lower learning, whereas higher intelligence was linked to higher learning and also to lower stress. Hence, our findings again support the often assumed benefits of tests—even when simultaneously considering learners’ intelligence and and when considering the by tests caused stress perceptions. Notably, controlling for stress further increases these long-term learning benefits. We then discuss some limitations and boundaries of our work as well as ideas for future studies.

## Introduction

The following work raises the question if normally beneficial learning tests actually serve as double-edged swords, thus, if they can result in both beneficial as well as detrimental effects: More specifically, the present work was conducted to simultaneously focus on the often observed positive long-term learning effects of tests as difficult and demanding learning strategies (see, e.g., Adesope et al., 2017; Yang et al., 2021) but also on potential negative (side) effects caused by such learning tests, namely, increased stress or anxiety perceptions (see, e.g., Hinze and Rapp, 2014; Wenzel and Reinhard, 2021). Such increased stress perceptions should have further detrimental effects on learning in general as well as on the beneficial effects of tests on long-term learning in specific (see, e.g., Seipp, 1991; Hinze and Rapp, 2014). Additionally, because recent studies indicated that higher intelligence is valuable for the effectiveness of tests (see, e.g., Minear et al., 2018; Wenzel and Reinhard, 2019), the present work also investigates if higher intelligence moderates the benefits of tests, thus serving as a prerequisite or boundary condition. In line with this, different previous studies indirectly supported the assumption that intelligence might also act as a buffer for negative effects of tests on immediate stress perceptions (see, e.g., LePine et al., 2004; Abín et al., 2020) and for the detrimental effects of stress perceptions on learning outcomes (see, e.g., Chuderski, 2014; Reeve et al., 2014). Hence, the present work bridges different research fields and simultaneously focuses on beneficial and detrimental effects of tests as well as on potentially moderating effects of intelligence as an important individual difference. Simultaneously testing these different research issues seems necessary for being able to give empirically well-grounded advice regarding the application of tests in university or school settings to learners and lecturers alike—especially because we not only investigate learning outcomes but also students’ experiences and perceptions as well as individual differences as potential prerequisites.

More specifically, focusing on these research questions is extremely relevant due to the importance of successful and durable later learning outcomes in school and university settings. Notably, although difficult learning strategies, like tests, have often been shown to increase long-term learning compared to learning strategies that are more fluent and simpler, learners and lecturers mainly assume the contrary (e.g., Karpicke et al., 2009; Diemand-Yauman et al., 2011; Kornell et al., 2011; Dobson and Linderholm, 2015; Bjork and Bjork, 2019). Thus, learners normally regard easy and fluent learning strategies as more effective and most prefer simpler strategies, like repeated reading—and such misconceptions even stick with teachers-to-be (e.g., Book et al., 1983; Koriat and Ma’ayan, 2005; Karpicke et al., 2009; Bjork et al., 2015). Hence, it is extremely important to conduct further empirical work to be able to give well-grounded advice to learners and lecturers alike that—or if—difficult tests are helpful and should be applied in actual university learning settings. Otherwise, they might not apply such tasks on their own. In line with this, lecturers and teachers often express concerns about the effectiveness of such difficult learning strategies for all of their students (e.g., Diemand-Yauman et al., 2011; Lipowsky et al., 2015), which is why we also test the importance of (higher) intelligence as a prerequisite for the beneficial effects of tests. This is relevant as it could further specify for which group of learners tests are beneficial and for which they are not. We thereby choose intelligence as an individual difference because it was often cited as one of the strongest predictors for academic achievement and is generally strongly associated with varying operationalizations of successful human behavior (see, e.g., Bornstein et al., 2013; Strenze, 2015). Surprisingly, we could not find much research concerning potential moderating effects of intelligence on the effectiveness of tests for long-term learning outcomes. In addition, and apart from such later learning outcomes, we also focus on learners’ perceptions of tests to explore if these normally beneficial learning tasks also lead to negative side-effects like increased immediate stress perceptions during and directly after learning. This seems relevant because it is often argued that students’ experiences and perceptions of different situations are seldom the main focus of experiments (see, e.g., Edwards and Templeton, 2005)—even though stress perceptions include, among others, subjective distress, higher degrees of worry, emotionality, tension, anxiety, nervousness, pressure, intrusive and disturbing thoughts, feelings of overwhelm, and lack of confidence (see, e.g., Epel et al., 2018). Hence, such stress perceptions in themselves are extremely unpleasant and undesirable but were additionally often shown to lead to further negative consequences like reduced motivation, mood disturbances, or health problems (e.g., DeLongis et al., 1988; Hobfoll, 1989; LePine et al., 2004). In line with this, stress perceptions have often been shown to be associated with lower learning outcomes (e.g., Seipp, 1991), so that stress perceptions might even act as a mediator of the beneficial effects of tests on later learning outcomes. Notably, this would be completely inconsistent with the intention of using tests in schools or universities and should therefore be thoroughly explored. Thus, it is extremely important to know if tests—even those conducted as low-stakes learning situations—lead to negative consequences, like increased stress perceptions, and if these would, paradoxically, be linked to reduced benefits of tests. It is also important to determine whether these negative side effects of tests on stress perceptions and the detrimental effects of stress on later learning outcomes arise for all learners or only for those with lower cognitive abilities. Hence, we also test if intelligence moderates these effects, thus, if immediate stress perceptions caused by tests or detrimental effects of stress perceptions on later learning outcomes decrease with higher intelligence. This would indicate that intelligence might also serve as a protective factor for potentially negative side effects caused by such learning tests and for detrimental effects of acute stress perceptions. In turn, such findings might further help to specify for whom tests are actually desirable. Taken together, focusing on and answering these research questions is very important regarding potential advice for teachers and lecturers concerning the utilization and practical application of learning tests in schools and universities. We further think that the present work focuses on new and extremely relevant issues while also trying to replicate previous findings (e.g., the benefits of tests as well as increased stress perceptions due to tests) that are of great relevance for the research field. Moreover, to our knowledge, no previous studies were conducted to test these assumptions, and none simultaneously tested prerequisites, beneficial effects, and potentially detrimental effects of tests. Hence, we want to highlight these important issues and stimulate future research. In the following, we want to start with presenting a state of the art literature overview regarding our posed research issues.

### Tests As Desirable Difficulties for Learning

Due to the importance of learning, knowledge acquisition, and academic achievement, a lot of researchers investigated varying learning strategies that improve durable long-term learning: For instance, *desirable difficulties* as challenging, demanding, and non-fluent learning processes have often been found to enhance later long-term learning outcomes compared to easier and more fluent learning processes (e.g., Bjork, 1994; Karpicke et al., 2009; Bjork and Bjork, 2011, 2020). Thus, although these effortful learning strategies appear to slow the learning process down at first and cause difficulties and challenges for learners, they increase information processing, retrieval, transfer, and ultimately leaners long-term learning (e.g., Bjork and Bjork, 2011, 2019, 2020). The term desirable difficulties thereby acts as an umbrella term for different intentionally hindered learning strategies, which lead to beneficial effects for later long-term learning outcomes: These include, for instance, *disfluency* (using harder-to-read fonts; Diemand-Yauman et al., 2011) and *generation* (generating materials and solutions instead of passive consumption; Bertsch et al., 2007). One especially robust desirable difficulty is the application of tests (also: *testing, testing effect, retrieval practice, test-enhanced learning, and learning/practice tests*): Taking (learning) tests on previously studied materials increases long-term learning compared to easier and more passive re-reading tasks or compared to note-taking as a stronger control task—even concerning a multitude of difficult, complex, and curricular subjects in realistic learning contexts (e.g., McDaniel et al., 2007; Dunlosky et al., 2013; Rowland, 2014; Karpicke and Aue, 2015; Adesope et al., 2017; Batsell et al., 2017; Rummer et al., 2017; Yang et al., 2021). These beneficial effects of tests were, among others, found for different types of learning materials (e.g., factual information, vocabulary, conceptual information, longer scientific textbook paragraphs, traditional (live) lectures/lessons, and recorded e-lectures/video-presentations) and for different types of test questions (e.g., multiple-choice questions, short-answer questions, fill-in-the-blank questions, comprehension-based questions, application-based questions, transfer questions, and inferences; e.g., Roediger and Karpicke, 2006; McDaniel et al., 2011, 2013; Dunlosky et al., 2013; Rowland, 2014; Khanna, 2015; Jing et al., 2016; Adesope et al., 2017; Iwamoto et al., 2017; Heitmann et al., 2018; Feraco et al., 2020; Yang et al., 2021). Moreover, tests were beneficial in varying (face-to-face or online) settings (e.g., laboratories, universities, classrooms, and at home/outside of class) and for students of different age groups (e.g., elementary school students, high school students, and university students; e.g., McDaniel et al., 2007, 2011; Roediger et al., 2011; Rowland, 2014; Adesope et al., 2017; Yang et al., 2021). Notably, the benefits of tests were also shown to arise when tests were administered in varying (conventional, computerized, or technological) modalities (e.g., paper-pencil tests, orally delivered tests, tests administered with computers, tests administered on online-websites, tests using clicker response systems, tests applied with mobile devices, and tests conducted with online applications like Kahoot; see, e.g., McDaniel et al., (2013), Grimaldi and Karpicke, (2014), Feraco et al., (2020), Wang and Tahir, (2020), Yang et al., (2021). Thus, researchers often recommend the application of tests as an effective learning task to increase learners long-term learning outcomes.

Theoretically, these beneficial effects of tests are often attributed to the stimulation of cognitive processes that increase the understanding, deeper semantic/cognitive processing, and encoding of information (e.g., Bjork, 1994; Bjork and Bjork, 2011; Dunlosky et al., 2013; Rowland, 2014). Tests are also supposed to lead to more analytic and elaborative thinking, more (effortful) retrieval practice, better anchoring of the learned information in long-term memory, and to an allocation of more effort and more cognitive resources while learning (e.g., Bjork and Bjork, 1992, 2011; Dunlosky et al., 2013; Rowland, 2014). Most important, the beneficial effects of tests are often argued to be stronger when the applied tests are more difficult and thereby elicit more difficult retrieval practice, when the test questions increase the depth of the required retrieval, and when learners have to indulge in more effort to work on and to solve the test questions (e.g., Tyler et al., 1979; Alter et al., 2007; Pyc and Rawson, 2009; Rowland, 2014; Maass and Pavlik, 2016; Greving and Richter, 2018). Tests were also shown to be more beneficial the more information learners were able to successfully retrieve and the more test questions they could answer correctly (e.g., Richland et al., 2005; Rowland, 2014). In line with this, previous work also yielded that desirable difficulties only increase long-term learning for learners who possess sufficient cognitive resources (e.g., higher working memory capacities), further knowledge (e.g., background/prior knowledge, experience, and expertise), special skills (e.g., higher reading skills), or for those that were generally high achieving (e.g., McNamara et al., 1996; Kalyuga et al., 2001; McDaniel et al., 2002; Carpenter et al., 2016; Lehmann et al., 2016). McDaniel et al. (2002) thereby argued that even when learners can correctly solve difficult generation tasks, this consumed a lot of their processing capacities. This is why only more able readers—and not less able readers—benefitted from generation tasks: Only these learners still had cognitive capacities left to further process and deeper encode the generated information after solving the difficult tasks. Notably, these findings and argumentations indicate that desirable difficulties—and especially tests—have to be difficult, demanding, and taxing to be beneficial but that learners must simultaneously be sufficiently equipped to master these posed challenges, must possess the skills to successfully respond to the difficult tasks and to successfully retrieve information, and must be able to muster the needed increased effort (e.g., Richland et al., 2005; Bjork and Bjork, 2011, 2019; Kornell et al., 2011; Alter et al., 2013; Oppenheimer and Alter, 2014; Rowland, 2014; Karpicke, 2017; Kaiser et al., 2018). This, however, may not prove possible for every learner—but should apply to leaners with higher intelligence.

### Tests and Intelligence

Intelligence has often been shown to be one of the strongest predictors for long-term learning, information retrieval, or academic achievement, and it is also argued to be especially valuable and predictive for difficult and stimulating learning environments and complex materials (e.g., Gottfredson, 1997; Kuncel et al., 2004; Fergusson et al., 2005; Bornstein et al., 2013; Roth et al., 2015; Stadler et al., 2015; Stern, 2015, 2017; Strenze, 2015). Moreover, intelligence is even defined as the ability to learn, to reason, and to solve problems and has also often been found to be associated with successful information processing, successful retrieval from long-term memory, and higher working memory capacities (see, e.g., Gottfredson, 1997; Sternberg, 1997; Oberauer et al., 2005; Bornstein et al., 2013; Stern, 2015, 2017; Wang et al., 2017). Hence, taken together, higher intelligence is not only generally important for long-term learning outcomes but also seems to be fundamental for tests to be actually beneficial and for learners to be actually able to reap those benefits. Thus, intelligence should moderate the beneficial effects of tests, insofar as that especially learners with sufficient cognitive abilities and higher intelligence should benefit from desirable difficulties and tests, particularly when learning with complex and curricular materials: Such learners should be able to successfully retrieve, further process, and understand the learned information and to manage such difficult tests without being cognitively overwhelmed—even after working on difficult and cognitive capacities reducing tasks (e.g., Kalyuga et al., 2001; McDaniel et al., 2002; Lehmann et al., 2016). Two previous studies found supporting evidence for the assumption that intelligence moderates the beneficial effects of tests: First, a study from Minear et al. (2018) yielded that higher fluid intelligence increased the positive effects of tests for difficult, as opposed to easy, information (regarding Swahili-English word pairs; learners with lower fluid intelligence showed the reverse effect). Second, Wenzel and Reinhard (2019) found that only at least averagely intelligent learners achieved higher long-term learning in a test condition compared to averagely intelligent learners in a re-reading control condition. Relatively intelligent learners (intelligence one standard deviation above mean) profited even more from difficult tests (Wenzel and Reinhard, 2019). Hence, these argumentations and findings imply that special prerequisites, like average or higher intelligence, must be given so that learners can even reap the benefits of tests. However, contrary findings also exist (showing different or no interactions between intelligence and the effectiveness of tests, e.g., Brewer and Unsworth, 2012; Robey, 2017), so that further work is still valuable.

Interestingly, the findings of Wenzel and Reinhard (2019) also highlighted that relatively unintelligent learners (intelligence one standard deviation below mean)—albeit they indulged in more effort and suffered a more strenuous and demanding way of learning—did not outperform less intelligent learners that instead studied with easier, more fluent, and less demanding re-reading tasks. Thus, the learning outcomes of less intelligent learners in both learning conditions did not differ from each other, whereas learners’ subjective experiences and perceptions during learning should have differed strongly. This in turn raises the question if further factors additionally to or beyond long-term learning must be considered when contemplating whether or not to apply tests in school or university settings. For instance, difficult learning tasks were previously shown to increase perceptions of threat or anxiety, experiencing difficulties as well as giving incorrect answers was found to feed negatively into self-perceptions, and performing poorly increased stress perceptions (e.g., O’Neil et al., 1969; Schunk and Gaa, 1981; Sarason and Sarason, 1990). Difficult learning tasks and tasks that require more effort, more time, and more workload were additionally often perceived as more stress-inducing compared to easier tasks (e.g., Kausar, 2010). Thus, tests might result in negative (side) effects like increased stress perceptions (which would be especially undesirable if the respective learners did not even profit from taking such tests).

### Tests and Perceptions of Stress or Anxiety

According to the *transactional theory of stress* (e.g., Lazarus and Folkman, 1987), perceptions of stress or anxiety arise when working on tasks (or when being in situations) that are perceived as threatening instead of challenging and in which individuals think that they do not possess enough resources or enough cognitive abilities to cope with the posed demands. Perceived imbalances between difficult tasks and learners’ own capabilities or resources also result in stress perceptions (see, e.g., McGrath, 1970; Lazarus, 1990; Kausar, 2010). Unsurprisingly, most students experience test situations, especially (graded) final high-stake tests, (summative) exams, or (competitive) school entrance examinations, as stressful, pressuring, and unpleasant (e.g., Sarason, 1984; Beilock, 2008; Bradley et al., 2010; Jamieson et al., 2016; Leiner et al., 2018). It was also observed that the majority of students’ academic stress stems from taking and studying for exams and from getting examination results (see, e.g., Abouserie, 1994). However, apart from such (graded) examinations, even tests solely used as learning situations might be stress-or anxiety-inducing—because tests as desirable difficulties must even per definition be challenging, effortful, and difficult, and might thus be perceived as overwhelming. In line with these assumptions, Hinze and Rapp (2014) conducted a laboratory study using science texts as study materials and applied re-reading tasks, low-stakes learning tests, or high-stakes learning tests. Stakes were thereby operationalized through instructions given before the learning tests stating that monetary rewards for the learner and a fictive partner were either independent of learners’ later final test results or dependent of their later final test results. The authors found that even low-stakes tests led to more immediate feelings of pressure than re-reading tasks and that high-stakes tests further led to more state anxiety than low-stakes tests and re-reading tasks (notably, these results were independent of participants’ trait anxiety and there were no interactions between the learning condition and trait variables, Hinze and Rapp, 2014). Another laboratory study also found that learning situations including a short test (on mathematical concepts and materials) were evaluated as more negative and as more stress-and anxiety-inducing than learning situations including a reading control task (these findings were also independent of participants’ trait stress or trait anxiety; Wenzel and Reinhard, 2021). Interestingly, contrary results were also found (see, e.g., Agarwal et al., 2014; Nyroos et al., 2016) and even though these can be explained due to methodological differences, replications are still advantageous. Apart from that, it is furthermore possible that these effects of tests on stress perceptions do not arise for learners with higher intelligence and that intelligence might moderate these negative effects.

### Intelligence and Perceptions of Stress or Anxiety

Because learners with higher intelligence should generally be able to solve difficult tasks and to answer more test questions successfully, they should, in turn, perceive tests as less threatening, less stressful, less difficult, less overwhelming, and thus as more manageable than learners with lower intelligence. In line with these assumptions, previous work showed that cognitive abilities were negatively correlated to situational stress experiences, math anxiety, state anxiety, and to ratings of difficulty of varying learning tasks (e.g., Efklides et al., 1997; LePine et al., 2004; Abín et al., 2020). Students that were extremely high-achieving in mathematics were also less math anxious, were more motivated, had more self-efficiency, and reported more enjoyment while learning (e.g., García et al., 2016). A study from Goetz et al. (2007) fittingly yielded that emotions experienced by school students during a mathematics achievement test differed based on their abstract reasoning abilities: Anger and anxiety were more prominent for students with lower abilities, whereas enjoyment was more prominent for students with higher abilities. However, if stress nonetheless arises due to tests, such generally unpleasant perceptions are also associated with even further detrimental effects and lower learning outcomes.

### Effects of Stress and Anxiety on Learning Outcomes

For instance, higher stress and anxiety were often found to be linked to lower motivation to learn, more errors, lack of concentration, disruptions in attention, higher cognitive load, and reduced effort and persistence while learning (e.g., LePine et al., 2004; Chen and Chang, 2009; Kurebayashi et al., 2012). Anxiety and stress were also negatively correlated with cognitive information processing, the effectiveness of retrieval practice, learning outcomes, academic achievement, and learners (test) performance—especially as the tasks, test questions, or information become more complex, more cognitive demanding, and more difficult (e.g., Hembree, 1988; Seipp, 1991; Struthers et al., 2000; Cassady, 2004a,b; Eysenck et al., 2007; Beilock, 2008; Chen and Chang, 2009; Khan et al., 2013; Sotardi et al., 2020). Hence, stress and anxiety were generally shown to have detrimental effects on learning outcomes but should further also negatively impact the normally beneficial effects of tests. In line with these assumptions, Mok and Chan (2016) found that highly test anxious participants in a learning test condition did not outperform participants in a re-reading control condition. Thus, there were no benefits of tests for highly anxious participants. Similar results were found by Hinze and Rapp (2014): High-stakes learning tests (operationalized through stating that monetary rewards were dependent of participants later final test results) increased pressure and state anxiety directly before the learning tests, which in turn decreased the benefits of these tests regarding later long-term learning. Only participants in a low-stakes learning test condition (in which monetary incentives were not stated to be dependent of participants’ test results) outperformed participants in the re-reading control condition. Hence, acute stress perceptions might mediate the beneficial effects of tests, insofar as that higher stress might partly diminish or even completely erase the beneficial effects of tests on long-term learning. Theoretically, such detrimental effects of stress on learning outcomes and on beneficial effects of tests are assumed to arise because stress and anxiety lead to worries and cognitive interference indicated by intrusive, distracting, and irrelevant thoughts. These, in turn, disrupt task-specific information processing, interfere with cognitive processes, impair retrieval, and divert the needed attention and focus away from the learned information, thereby depleting cognitive capacities and storage and processing resources: These consumed resources and capacities would otherwise have been needed for retrieving information, for successfully answering test questions, and for further processing, encoding, or decoding of these information (see, e.g., *attentional control theory, cognitive interference model, distraction theories, processing efficiency theory, and retrieval disruption hypothesis*; Eysenck and Calvo, 1992; Ashcraft and Krause, 2007; Eysenck et al., 2007; Hinze and Rapp, 2014; Sarason, 1984; Tse and Pu, 2012; however, contrary results and contrary theories also exist, showing, for instance, positive linear effects of stress on learning outcomes or non-linear/inverted U-shaped relations of anxiety and performance; see, e.g., LePine et al., 2004; Keeley et al., 2008; Sung et al., 2016). Notably, such detrimental effects of acute stress and anxiety on learning might again be less pronounced for learners with higher compared to learners with lower intelligence. Thus, intelligence might moderate these detrimental effects.

### Intelligence and Detrimental Effects of Stress and Anxiety

Because higher intelligence is generally linked to better information processing, higher (working memory) capacities, and better retrieval from long-term memory, learning outcomes of more intelligent learners should not be harmed (as strongly) by stress perceptions, worry, or reduced cognitive capacities compared to learning outcomes of less intelligent learners (e.g., Oberauer et al., 2005; Stern, 2015, 2017; Wang et al., 2017). Thus, such learners should still possess enough resources and capacities to successfully work on difficult tasks and to further process the retrieved and studied information even after perceiving stress. In line with this, researchers assumed that higher domain-specific abilities or extra processing resources should be able to compensate detrimental effects on learners’ initial acquisition of information and on their later learning outcomes caused by stress and anxiety (e.g., Tobias, 1984; Naveh-Benjamin, 1991; Eysenck and Calvo, 1992; Eysenck et al., 2007). Fittingly, a study from Tse and Pu (2012) found that less effective and less successful retrieval practice caused by higher test anxiety could be compensated by higher working memory capacities. Thus, anxiety had only detrimental effects for learners with lower working memory capacities (see also Ashcraft and Krause, 2007; Johnson and Gronlund, 2009; Owens et al., 2014; for contrary results, see Beilock, 2008). Previously conducted work also yielded that cognitive abilities had a buffering effect for negative consequences of distraction, insofar as that distraction only had a detrimental effect on (exam) performance for lower ability learners but did not decrease performance of higher ability learners (Reeve et al., 2014). It was furthermore shown that (fluid) intelligence moderated the impact of state anxiety on working memory functioning: The negative impact of state anxiety on working memory functioning was shown to diminish with higher intelligence and anxiety only negatively affected working memory for learners with intelligence below median (Chuderski, 2014).

### The Present Research

Taken together, the present research simultaneously focused on tests as desirable difficulties, their beneficial effects on later learning outcomes, and their negative effects on stress perceptions. We further focused on detrimental effects of increased stress on later learning outcomes and on the normally beneficial effects of tests. Moreover, we also explored learners’ intelligence as a potential prerequisite for beneficial effects of tests as well as potentially moderating effects of intelligence: Higher intelligence should increase beneficial effects of tests on later learning outcomes, decrease stress perceptions caused by tests, and reduce detrimental effects of stress on learning.

**Figure 1:**
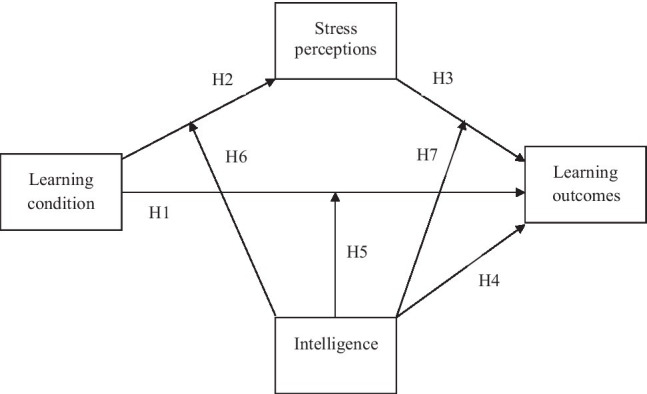
Graphical depiction of the different variables and the assumed hypotheses. The learning condition consists of a re-reading control condition (0) and a test condition (1).

Following the in the Introduction presented empirical and theoretical argumentations, we thereby suppose the following hypotheses (see Figure 1 for a graphical depiction). For a better comprehensibility, we want to sort the hypotheses according to main and interaction effects: First, we assume that tests, compared to re-reading tasks, result in beneficial effects on later learning outcomes: Thus, a test condition should lead to higher later learning outcomes than a re-reading control condition (*Hypothesis 1*). Nonetheless, working on tests should also increase acute stress perceptions compared to working on the re-reading task (*Hypothesis 2*). In turn, such acute stress perceptions were assumed to be negatively correlated with participants later learning outcomes (*Hypothesis 3*). In that regard, we assumed that acute stress perceptions would mediate the effect of the learning condition (and thus the beneficial effects of tests) on later learning: Higher stress perceptions caused by tests should be linked with reductions of the normally beneficial effects of tests on later learning outcomes. Moreover, we assume intelligence to be positively correlated with later learning outcomes (*Hypothesis 4*).

We also assumed the following three interaction effects: First, we assumed that the beneficial effects of tests on later learning outcomes should be moderated by participants intelligence: Beneficial effects should be stronger for more intelligent participants and weaker for less intelligent participants (*Hypothesis 5*). Second, the negative effects of tests on stress perceptions should also be moderated by intelligence: More intelligent participants should perceive less acute stress when learning with a test than less intelligent participants in the test condition (*Hypothesis 6*). Third, the detrimental effects of stress perceptions on later learning outcomes should also be moderated by intelligence: Later learning outcomes of more intelligent participants should be less harmed by stress perceptions than later learning outcomes of less intelligent participants (*Hypothesis 7*).

To test these hypotheses, we conducted a laboratory study consisting of two sessions. We therefore designed a realistic learning situation that could be easily transferred to actual universities or schools. We used, for instance, complex and curricular learning materials that are actually applied in university courses. Thus, we tried to replicate the often found beneficial effect of tests (compared to easier and more passive re-reading control tasks) for difficult and realistic materials. We also conducted a short learning test, including varying test questions formats (e.g., short-answer and multiple-choice questions) that students should often encounter in their university lives (e.g., at the end of textbook chapters, in examinations, …). Moreover, to reliably investigate whether learning tests actually lead to stress perceptions, we devised an extremely low-stakes learning test situation that still resembled an actual university course as closely as possible. Hence, we did not want to experimentally manipulate stress but wanted to observe if stress perceptions would even occur in virtually pressure-less learning situations that either include a short test task or a re-reading task. Fittingly, we only instructed participants to do their best while learning and did include neither monetary rewards (see, e.g., Hinze and Rapp, 2014) nor grades (see, e.g., Khanna, 2015) as further incentives that might influence their perceptions and evaluations of these learning tests. This also ensured that our laboratory learning situation would resemble a typical learning situation in university or school settings. To further ensure that the test task would be without stakes or artificial stressors, we avoided using learning materials that might be stress-or anxiety inducing in themselves (like mathematical or statistical information; see, e.g., Wenzel and Reinhard, 2021) and applied a test in which participant did not even have to say their answers out loud in front of their peers (contrary to Wenzel and Reinhard, 2021; see also England et al., 2017). To adequately assess participants stress perceptions caused by the learning situation, we measured their state stress directly after they completed the respective learning task and explicitly instructed them to refer to their perceptions and experiences while learning (contrary to previous work where stress was assessed, for instance, before participants worked on the respective tests, after the tests but with a longer delay, or even retrospectively at the end of the academic year; see, e.g., Agarwal et al., 2014; Hinze and Rapp, 2014; Nyroos et al., 2016). Finally, we must note that our work was planned and conducted shortly before the onset of the COVID-19 pandemic. Therefore, our theoretical and methodological considerations mostly focused on conventional learning settings or conventional learning modalities that were rather typical for our respective university before the restrictions due to COVID-19 were implemented. This includes, for instance, face-to-face learning situations in which students learn alongside their peers with a lecturer present as well as directly in-class implemented learning tasks (see, e.g., Yang et al., 2021, for the benefits of supervised in-classroom tests compared to tests administered outside of classrooms). Hence, our laboratory setting was intended to mirror a typical learning situation before most education was transferred to distance e-learning.

## Materials and Methods

### Participants

Power was set to 0.90, and sample size was calculated to detect a medium effect (*f=* 0.25).^1^ Using G^*^Power (Faul et al., 2009), a power analysis revealed a needed sample size of *N*=171 to detect a significant effect (alpha level of 0.05)—given there is an effect (regrettably, we later realized that—following the argumentation of Blake and Gangestad (2020)—this calculation would have already resulted in an underpowered sample size regarding the assumed interaction effects). Unfortunately, due to the COVID-19-outbreak and later lock-down restrictions, we also had to stop our recruitment and could not continue to collect data in the laboratory (this, in turn, further drastically reduced the power of our work, especially regarding the assumed interaction effects that are extremely underpowered). Due to this stop of our recruitment, our sample consisted of only 91 participants, from which two participants had to be excluded because they did not participate in both sessions of the study. Hence, our final sample consisted of *N*=89 participants (*M_age_*=24.18, *SD_age_*=6.25, range: 18–48; 70.8% female; 85.4% German native speakers). Of these, 96.7% were students at a German university. Seventy-three of them (82.00%) studied psychology, and the remaining studied, among others, architecture, education, philosophy, social science, languages, and politics. Each participant was randomly assigned to one of the two between-subjects learning conditions: the re-reading control condition (*n*=47) or the test condition (*n*=42). Before starting, each participant had to provide their approval through reading and agreeing to a written informed consent. The study was conducted in full accordance with the Ethical Guidelines of the DGPs and the APA, and the funded project was approved by the Ethics Committee affiliated with the funding source.

### Procedure

Up to seven participants could simultaneously take part in our study. On average, 3.83 students participated simultaneously (*SD*=1.97, range=1–7). For less diversion and more anonymity, each participant sat in a workplace with dividers in front of a computer. All tasks were complete on this computer. In general, participants arrived together, started the study together, and worked simultaneously on the specific tasks but did not directly communicate with each other while undergoing the study and while learning. Apart from a brief welcome from the experimenter, short instructions when different tasks were supposed to start and stop, and a short farewell (all oral instructions were read out loud from standardized texts), all materials and all instructions were presented on the computer. The experimenter (the first author) otherwise only stopped the time for time-limited tasks, made sure that these time limits were met, and monitored that participants generally adhered to the instructions (e.g., the experimenter sometimes reminded participants to further work on the specific learning tasks if participants had stopped working although they still had time left for studying).

#### Session 1

After a brief welcome and after reading and agreeing to the written informed consent, participants’ demographic measures were assessed (e.g., age, gender, occupational status, native language, ethnicity, field of study, and graduation grade). Thereafter, we measured an intelligence estimate using a 3-min intelligence screening (*mini-q*; Baudson and Preckel, 2015; based on Baddeleys *verbal reasoning*, Baddeley, 1968; further: *intelligence-estimate*). The mini-q is a reliable and valid screening instrument for general (fluid) cognitive abilities that accurately assesses speeded reasoning as a conglomerate of reasoning, abstract thinking, and processing speed (Baudson and Preckel, 2015). The mini-q includes 64 tasks that each consist of a statement describing three geometrical figures (square, triangle, and circle) that participants have to declare as right or wrong (for two example items, see Baudson and Preckel, 2015) and have 3min to solve as many of the tasks as possible.^2^ Using a standard table including a representative adult sample, the sums of correctly solved tasks can then be transformed to estimations of intelligence scores (*M*=100, *SD*=15). Participants were generally instructed to try their best while working on these task. To ensure that our instructions would not frame the task as needles pressuring or stressful, we correctly described that the task focused on participants reasoning and abstract thinking abilities but did not explicitly highlight that it thereby also serves as an intelligence-estimate. This was done because previous work sometimes induced stress perceptions by explicitly presenting tasks as intelligence tests or by using instructions that generally increase participants’ expectations of having to work on demanding or threatening intelligence tests (see, e.g., Kimmel and Bevill, 1985; Zeidner, 1998).

Before the learning phase started, we then informed participants that we wanted to explore the effectiveness of different learning tasks, which is why it would be important that they give their best while learning and that they should imagine to be studying for one of their actual university courses. We also reminded them that the ability to quickly and successfully learn new information is extremely advantageous in their everyday university lives and asked them to learn as intensively as they normally would. Participants were also informed that they would, 1week later, be charged with taking a final test covering the learned information. The learning materials consisted of one textbook chapter describing the brain’s lateralization based on a standard introductory textbook that is often adopted for university courses in biopsychology (Pinel and Pauli, 2012). Thus, the learning material was difficult, complex, and curricular. Before participants initially read the text, we assessed their prior knowledge regarding this topic to check if it differed between participants in the two learning conditions. We thereby implemented three open-ended questions (e.g., *Which function is linked to the Broca area?*) that participants answered within 3min.

In the following *first learning phase*, all participants once read the three textbook pages concerning the brain’s lateralization as an initial study opportunity. They were therefore given about 10min. For the subsequent 10min of the *second learning phase*, each participant was then (*via* the computer they worked on) randomly and individually assigned to either the re-reading control condition or to the test condition.

##### Re-Reading Control Condition

In the re-reading control condition, participants were again presented with the textbook chapter. They were instructed to read the text as often as they wanted in the given time and to learn, understand, and memorize the information.

##### Test Condition

In the test condition, participants were presented with a learning test inquiring different aspects of the previously read textbook. The test consisted of 17 questions. These were multiple-choice questions and open-ended questions, which required both short answers consisting of single words or bullet points as well as longer, more detailed answers (participants could gain up to 2 points per correct answer; a maximum of 20 points could be gained; for examples, see Appendix A).

Following, participants state stress caused by the learning condition was measured with the German version of the Perceived Stress Questionnaire (PSQ; Fliege et al., 2001; based on Levenstein et al., 1993) using 20 items (*α*=0.89; e.g., *You felt tense*) on a four-point Likert-like scale from one (*almost never*) to four (*usually*). To assess participants immediate stress perceptions, they were explicitly instructed to refer their ratings to their perceptions and experiences during the just finished second learning phase.

Participants then answered some manipulation check questions regarding the second learning phase, e.g., regarding the difficultly, strenuousness, or helpfulness of the learning task, their assumed success, as well as their evaluations of the second learning phase as negative/positive and challenging/threatening (e.g., *How difficult did you find working on the second learning phase?* one (*very easy*) to five (*very difficult*); see Appendix A for all 6 manipulation check questions). Thereafter, participants in the test condition received feedback in form of an answer sheet displaying the correct answers to the test questions. Finally, participants were asked if they had already known the learning materials or the applied intelligence screening and were instructed not to study the learned materials in the meantime.

#### Session 2

In the second session (1week after Session 1; *M_days_*=7.12, *SD_days_*=0.50, range: 7–10), participants later learning outcomes were assessed. Therefore, participants were required to work on a final test for 10min. The final test included 21 questions (participants could gain up to 2 points per correct answer; a maximum of 27 points could be gained). In line with the learning test in Session 1, the final test consisted of multiple-choice and open-ended questions. Eight of the final test questions were identical to questions previously used in the learning test, while seven of them were slightly changed to assess transfer. The remaining six final test questions asked about information that were part of the read textbook chapter but had not been previously implemented in the learning test in Session 1.

In the end, participants were asked if they had re-studied the learning materials in the interim. They were then shortly debriefed and received the opportunity to take part in a raffle for a total of 200 Euro. Psychology students could alternatively earn course credit.

## Results

Participants’ age, gender distribution, native language distribution, graduation note, the number of students that participated simultaneously, the time lag between Sessions 1 and 2, participants’ intelligence-estimate scores, and their prior knowledge did not significantly differ between the test condition and the re-reading control condition (all *p*s≥0.163). This indicated that the random distribution of participants to the two conditions had been successful. Comparing the manipulation check questions between participants in the test condition and participants in the re-reading control condition indicated that the manipulation of the conditions had also been successful: Most important, participants in the test condition rated the learning situation as significantly more difficult than participants in the re-reading control condition, *M_re-reading_*=2.11, *SD_re-reading_*=0.96, *M_test_*=2.90, *SD_test_*=1.12, *t*(87)=−3.62, *p*=0.001, *d*=−0.76 (95% CI[−1.20; −0.32]). The effect size can be classified as medium to high. The test condition was also evaluated as slightly more challenging than the re-reading control condition, *M_re-reading_*=2.74, *SD_re-reading_*=0.57, *M_test_*=2.26, *SD_test_*=0.83, *t*(87)=3.23, *p*=0.002, *d*=0.68 (95% CI[0.25; 1.11]). There were no significant differences between ratings of strenuousness, helpfulness, overall (positive or negative) evaluation, and successfulness of the two learning conditions (all *p*s≥0.081).

Descriptively, participants achieved on average an intelligence-estimate score of 112.03 (*SD*=16.21, range: 73–154). Their average state stress score was 2.09 (*SD*=0.52, range: 1.20–3.70). Considering the final test measuring their later learning outcomes, participants were on average able to give 13.84 of 27 (51.26%) correct answers (*SD*=4.33, range: 4–24).

To test Hypothesis 1, we conducted a *t*-test to compare participants later learning outcomes in both learning conditions: *M_re-reading_*=12.87, *SD_re-reading_*=4.17, *M_test_*=14.93, *SD_test_*=4.30, *t*(87)=−2.29, *p*=0.025, *d*=−0.49 (95% CI[−0.92; −0.06]). As assumed, participants in the test condition answered more final test questions correctly than participants in the re-reading control condition, serving as first support for Hypothesis 1. The size of this effect can be interpreted as medium.

Following, we conducted another *t*-test to compare participants’ acute stress perceptions in both learning conditions to test Hypothesis 2: *M_re-reading_*=1.99, *SD_re-reading_*=0.49, *M_test_*=2.21, *SD_test_*=0.52, *t*(87)=−2.04, *p*=0.045, *d*=−0.44 (95% CI[−0.87; −0.01]). Supporting Hypothesis 2, participants in the test condition perceived more state stress during and immediately after the learning situation compared to participants in the re-reading control condition. The size of this effect can be classified as small to medium.

In turn, such stress perceptions were significantly and negatively correlated with later learning outcomes {*r*=−0.26 (95% CI[−0.44; −0.06]), *p*=0.014},^3^ showing a small to medium correlation. Thus, higher stress perceptions were linked to lower later learning outcomes indicated by fewer correctly solved final test questions. This served as first support for Hypothesis 3.

To test whether the beneficial effects of tests on later learning outcomes were mediated by participants acute stress perceptions, we then ran a mediation analysis^4^ with Process (model 4; Hayes, 2018). Thus, we tested direct effects of the learning condition on participants later learning outcomes and indirect effects of the learning condition on participants later learning outcomes *via* state stress (all predictors and the potential mediator were z-standardized; see Figure 1 for a graphical illustration of these assumed relations and our hypotheses). The learning condition significantly predicted participants perceived stress during the learning situation (path a), *B*=0.43, *SE*=0.21, *t*(87)=2.03, *p*=0.045. Thus, tests increased acute stress perceptions, which served as further evidence for Hypothesis 2. In turn, such state stress predicted participants later learning outcomes (path b), *B*=−1.41, *SE*=0.39, *t*(86)=−3.60, *p*=0.001. Thus, higher stress perceptions were linked to lower later learning outcomes, serving as further evidence for Hypothesis 3. We also found a significant total effect (path c) of the learning condition on later learning outcomes, *B*=2.06, *SE*=0.90, *t*(87)=2.28, *p*=0.025. The direct effect (path c’) of the learning condition on later learning outcomes (when simultaneously controlling for participants’ stress perceptions) was also significant, *B*=2.66, *SE*=0.88, *t*(86)=3.04, *p*=0.003. Thus, we found the assumed beneficial effects of tests on later learning, which served as further evidence for Hypothesis 1. Moreover, the indirect effect of the learning condition on participants later learning outcomes *via* state stress was also significant (path a x path b), *B*=−0.60, 95% CI[−1.47; −0.04]. Notably, the direct effect was stronger than the total effect, showing that controlling for participants’ state stress increased the beneficial effects of the test condition. This indicated that state stress is not a mediator but a suppressor of the effect of the learning condition on later learning outcomes.

Furthermore, correlational analyses then showed that participants later learning outcomes were significantly correlated with their intelligence-estimates {*r*=0.34 (95% CI[0.14;0.51]), *p*=0.001, showing a medium correlation}. This served as first support for Hypothesis 4. Interestingly, the intelligence-estimate was also significantly—and negatively—correlated with participants state stress {*r*=−0.39 (95% CI[−0.55; −0.20]), *p*<0.001, showing a medium correlation}.

Finally, we conducted a moderated mediation analysis (Process, model 59; Hayes, 2018) to test all hypotheses—including the three assumed interaction effects (Hypotheses 5, 6, and 7)—simultaneously in a single statistical model (all predictors, the mediator, and the moderator were z-standardized; see Figure 1 for a graphical illustration of these assumed relations and our hypotheses). Because not all requirements were fulfilled (homoscedasticity was not given for one path of the mediation analysis, Breusch-Pagan test: *p*=0.031), we ran this analysis with heteroscedasticity robust standard errors imbedded in Process. Again, the learning condition significantly predicted participants perceived stress during the learning situation (path a), *B*=0.40, *SE*=0.20, *t*(85)=2.05, *p*=0.043. The intelligence-estimate was also a significant predictor for such stress perceptions, *B*=−0.34, *SE*=0.15, *t*(85)=−2.26, *p*=0.027. However, the intelligence-estimate did not moderate this negative effect of the learning condition on stress perceptions (learning condition*intelligence-estimate), *B*=−0.10, *SE*=0.19, *t*(85)=−0.55, *p*=0.586. Taken together, tests led to more acute stress perceptions than the re-reading control task, which again supported Hypothesis 2. Notably, although higher intelligence was generally linked to lower stress perceptions, the effect of the learning condition on stress perceptions was not moderated by the intelligence-estimate, thereby not supporting Hypothesis 5. Moreover, state stress, in turn, again predicted participants later learning outcomes (path b), *B*=−1.01, *SE*=0.50, *t*(83)=−2.04, *p*=0.045. The intelligence-estimate was, contrary to the previously conducted correlational analysis, not a significant predictor for later learning outcomes, *B*=1.16, *SE*=0.67, *t*(83)=1.73, *p*=0.088. The intelligence-estimate did also not moderate the detrimental effect of stress perceptions on later learning outcomes (stress perceptions*intelligence-estimate), *B*=−0.12, *SE*=0.54, *t*(83)=−0.22, *p*=0.829. Thus, higher stress perceptions were again linked to lower later learning outcomes, which again supported Hypothesis 3. However, intelligence neither predicted later learning outcomes nor moderated the detrimental effect of stress on later learning outcomes, hence, neither supporting Hypothesis 4 nor Hypothesis 6. Furthermore, there was a significant direct effect (path c’) of the learning condition on later learning outcomes, *B*=2.54, *SE*=0.85, *t*(83)=2.98, *p*=0.004. This effect was also not moderated by the intelligence-estimate (learning condition*intelligence-estimate), *B*=−0.10, *SE*=1.01, *t*(83)=−0.10 *p*=0.919. These findings again showed that tests were more beneficial for participants later learning outcomes than the re-reading control task and that this beneficial effect was independent of participants intelligence. This again supported Hypothesis 1 but not Hypothesis 7. The indirect effect of the learning condition on later learning outcomes *via* stress perceptions did also not differ depending on participants’ intelligence-estimates.

### Exploratory Analyses

Exploratory analyses can be found in Appendix B. These include, for instance, analyses focusing separately on the three different types of final test questions indicating later learning outcomes described in the methods section. We also depict correlations among participant ratings of the manipulation check questions (assessing their perceptions and evaluations of the two learning conditions) and participants stress perceptions.^5^

## Discussion

The present work was conducted to simultaneously test linkages among (learning) tests, acute stress perceptions, intelligence, and later learning outcomes (see Figure 1 for a graphical overview of our hypotheses). Addressing these linkages and testing our hypotheses is extremely relevant before tests—as potentially double-edged swords—are used in university and school settings. Summarizing, our results supported all assumed main effects (most effect sizes can thereby be categorized as small to medium) but none of the assumed interaction effects. In more detail, our data yielded that tests led to higher later learning outcomes 1week after the learning phase compared to the re-reading control condition. This fits the literature mentioned in the Introduction and again shows the benefits of applying tests as difficult learning tasks (e.g., Rowland, 2014; Adesope et al., 2017; Yang et al., 2021). However, also in line with our assumptions and the in the Introduction cited literature (e.g., Hinze and Rapp, 2014; Wenzel and Reinhard, 2021), the test condition also increased participants acute stress perceptions during and directly after learning compared to the re-reading condition. Although the descriptive statistics of stress perceptions were not extremely high (midpoint of the scale=2.00, *M_re-reading_*=1.99, *M_test_*=2.21) and the size of the effect was only small to medium, our results showed that even low-stakes learning tests were perceived as more demanding, more threatening, and more stressful than re-reading of previously studied materials. In turn, such stress perceptions were then negatively linked to later learning outcomes, thus supporting previous work that also reported detrimental effects of stress and anxiety on learning (e.g., Seipp, 1991; Hinze and Rapp, 2014; Sotardi et al., 2020). Interestingly, such increased stress perceptions served as a suppressor of the beneficial effects of tests on later learning outcomes (a mediation analysis found an indirect effect of the learning condition on long-term learning *via* stress perceptions): The direct effect of the learning condition controlling for stress perceptions was stronger than the total effect of the learning condition without controlling for differences in stress perceptions. Thus, the beneficial low-stakes test increased participants immediate stress perceptions and these triggered stress perceptions were in turn related to decreases of benefits of the test. Hence, although the test condition was still—albeit less—beneficial for later learning outcomes, it was even more effective when individual differences in stress perceptions were controlled for. Furthermore, as has often been shown before (see, e.g., Kuncel et al., 2004; Fergusson et al., 2005), higher intelligence was linked to higher achievement and higher later learning outcomes.^6^ Notably, higher intelligence-estimate scores were additionally related to lower stress perceptions in the learning situation. Thus, higher intelligence buffered feelings and perceptions of threat, demands, or pressure—which is also in line with literature cited in the Introduction (see, e.g., Efklides et al., 1997; LePine et al., 2004; Goetz et al., 2007). Nonetheless, intelligence did not moderate any of the main effects found in our study: The three hypotheses concerning interaction effects (learning condition*intelligence-estimate on stress perceptions, learning condition*intelligence-estimate on later learning outcomes, and stress perceptions*intelligence-estimate on later learning outcomes) were not supported by our data.

Two aspects of our sample were probably the main reasons that we were not able to support these hypothesized interaction effects: the intelligence-estimate scores of our participants and the size of our sample. Although the intelligence-estimate scores of our sample were normally distributed, participants had an average intelligence of 112.03 (*SD*=16.21, range=73–154), indicating that even the less intelligent participants in our sample were rather intelligent. In comparison, the relatively unintelligent learners that did not benefit from learning tests in the work of Wenzel and Reinhard (2019; Study 2) had intelligence scores lower than 86.39. In our sample, however, only three participants had intelligence scores that were lower than 86 (73, 84, and 85). Thus, we might have not been able to observe interaction effects due to these already relatively high intelligence scores. Even more important was, however, the small sample size of our work: As mentioned in our methods section, the sample size was—due to the COVID-19-outbreak and the resulting stop of our laboratory study—smaller than *a-priori* calculated (and the *a-priori* conducted and pre-registered sample size might erroneously have already been too small regarding potential interaction effects; see, e.g., Blake and Gangestad, 2020). Thus, it is most likely that the interaction effects were not detected because power was not sufficient.

All in all, even though not all our hypotheses were supported and although the sizes of the found effects can mostly be described as medium, our work raised important research issues and aims to serve as a first step to give (empirically well-grounded) advice to lecturers and teachers regarding the application of tests, their prerequisites, and their (positive as well as negative) consequences. Notably, the simultaneous testing of beneficial learning effects of tests, increased stress perceptions as negative (side) effects caused by tests, detrimental effects of such increased stress perceptions, and also potential moderating effects of learners intelligence has, to our knowledge, not been done before. Hence, our study highlights important research issues, uniquely contributes to the research field, and presents findings that are extremely stimulating for future work. Positively, we therefore conducted a laboratory setting that was similar to realistic learning situations in university settings (at least in this respective university and before the outbreak of the COVID-19 pandemic), insofar as that multiple students simultaneously worked on learning tasks with an experimenter present. Participants were thereby only instructed to learn as they typically would and to do their best without giving them further incentives to do well (like, e.g., monetary incentives that are normally not present in university settings). Moreover, the laboratory was set in a university building that hosts offices of lecturers as well as seminar rooms and many participants participated before or after their normal courses—hence, the setting of the study should have strongly resembled a typical university setting. Most important, the applied learning materials were complex and realistic materials that are actually applied in university courses and that are even—at least for most of the psychology students included in our sample—part of their curriculum. Regarding the test condition, we designed a short, realistic, low-stakes test, which included varying test question types (e.g., multiple-choice questions and short-answer questions requiring both shorter and longer answers) as well as varying levels of questions depths (e.g., asking for facts or asking for understanding, transfer, and application of the initially studied information). These test questions should closely resemble questions that are typically posed in university courses or that are included at the end of chapters found in many textbooks. Thus, our findings—indicating a benefit of short learning test that only require 10min of students’ time and that include varying complex test questions and difficult and curricular information—should be applicable and transferable to learning situations in actual universities and should not only be valid in laboratory settings. Hence, in line with previous work, we would advise lecturers to use the last 10min at the end of their courses to apply test questions concerning the contents of the respective lectures to help increase their students learning outcomes (this could be done, for instance, at the end of all or only some lecturers; see, e.g., Pashler et al., 2007; McDaniel et al., 2011; Iwamoto et al., 2017; Greving and Richter, 2018). Our work also indicates that such tests are beneficial for all university students independent of their intelligence and might, thus, be applied in different courses, different study paths, and for different educational backgrounds. However, our work also highlights negative (side) effects and detrimental effects caused by tests that lectures should consider and keep in mind when designing and using tests. Even though these effects were expected, they are still startling insofar as that the applied test was short, did not focus on excessively stress-inducing materials, and had no consequence for participants’ everyday lives. In line with this, participants worked on their own, did not have to say their answers out loud in front of their peers, and knew that their results would remain anonymous and that they only had to try their best without fearing consequences due to their performances (on, for instance, monetary incentives, grades, or general evaluations). Thus, although we conducted the test as a low-stakes learning situation in a laboratory setting without manipulating stress perceptions (and without choosing especially stressful tasks or information), the test nonetheless increased stress perceptions. This indicated that these found negative (side) effects of tests might be even more pronounced in actually relevant learning situations in schools or universities. Due to this assumption and due to the observed further detrimental effects of by tests caused stress perceptions on the beneficial effect of test, tests should be conducted as low-stakes and as stressless as possible—to optimize the benefits of tests on learning outcomes as well as to improve learners’ experiences and perceptions while learning. Thus, lecturers should try to implement tests that are at most similarly stress-inducing as the tests we applied in this work or try to design tests that are even less pressuring or threatening (without simultaneously reducing the difficulty of the test that is needed for the beneficial long-term learning effects of tests). For instance, previous work indicated that lectures might try to use more gamified learning strategies: Iwamoto et al. (2017), for instance, showed that short tests applied with Kahoot were beneficial for students learning outcomes and were even perceived and rated as positive by the respective students (see also Wang and Tahir, 2020 regarding the application of Kahoot, as well as Mavridis and Tsiatsos, 2017 for the application of game-based tests). The present work furthermore again showed the relevance of (higher) intelligence—albeit, it did not moderate any of the found effects—for cognitive variables like learning outcomes but also for affective variables like emotional reactions to potentially threatening situations. Although learners perceived tests as more stressful independent of their intelligence and although they similarly suffered under decreased learning outcomes due to higher stress perceptions independent of their intelligence, participants with higher intelligence still had some advantages compared to participants with lower intelligence, insofar as that higher intelligence was linked to less stress perceptions in both learning conditions.

Nonetheless, we have to note that our work is not without limitations, which is why the just described indications and applications should be considered with caution until further replications support our findings (especially regarding the conducted analyses testing the assumed interaction effects). Hence, we want to briefly discuss the limitations of our study as well as outlooks and ideas for future work. The most important limitation is, of course, that our sample size was smaller than *a-priori* calculated and that our work was therefore (especially regarding the assumed interaction effects) underpowered. Thus, future studies should in any case replicate our findings with a much bigger sample (see, e.g., Blake and Gangestad, 2020). Additionally, a large proportion (82.00%) of our participants studied psychology and were rather intelligent (*M*=112.03, *SD*=16.21). Thus, collecting a generally more diverse sample and a sample with more variance regarding participants’ intelligence scores is important for future work and for future replications—to ensure that the resulting findings are generalizable to different samples and to be able to give empirically well-grounded advice to lecturers and learners. The same applies to future replications using different (e.g., longer or multiple) tests, varying learning materials (e.g., regarding information that are definitely part of students curriculum and that are part of later graded examinations), or different (e.g., real university or school) settings. Future work could also focus more closely on potential impacts of different types of test questions on students’ perceptions or learning outcomes (see, for instance, Appendix B for exploratory analyses separating the in the present work applied three types of test questions). We also think that it would be valuable to conduct replications that try to control more strongly how participants in the re-reading control condition studied—hence, it is important to know if (and how often or how engaged) participants actually re-read the materials or if they simply skimmed through the text. Although the experimenter of our work reminded participants to keep reading if they had obviously stopped reading before the time limit was up, we unfortunately had no way of knowing if participants actually read the text, how often or how intensively they read the text, and if they thereby actually tried to understand and memorize the presented information. Thus, if participants only browsed through the text and did not genuinely re-read the text, this might have further increased the difference between the two learning conditions. Therefore, it would be advantageous if future work could focus even more on the re-reading control condition or if they could apply different, even stronger control conditions (e.g., note-taking). Additionally, longer delays between the learning phase and the later learning assessment would also be valuable to generalize our results found after 1week to longer delays and to more durable long-term learning effects. Furthermore, the future work could also use different or additional intelligence tests to focus even more on this important individual difference. Although the applied screening instrument serves as a reliable and valid estimation of general cognitive abilities as a conglomerate of reasoning, abstract thinking, and processing speed, it would still be advantageous to test whether the same results would arise when using longer, more general, or more complex intelligence measurements without short time limits. Chuderski (2014), for instance, stated that shorter and timed intelligence tests—which applies to the used intelligence screening—are often very similar to tests assessing working memory capacities. Thus, further replications would be valuable. Fittingly, future studies could also focus more closely on the assumed effects of intelligence on the benefits of tests to further investigate why or how these might arise: Should more intelligent learners, for instance, benefit more from tests because they are able to answers more questions successfully or because they can (independent of their actual success) better and deeper process the retrieved information and the solved answers? Apart from that, future work should also focus on ways to reduce stress perceptions caused by tests to maintain their benefits: For instance, researchers and lecturers could also test the application of emotion regulation techniques, coping strategies, online test formats, or repeated tests, and they could further prime the beneficial effects of tests or could generally try to modify learners’ perceptions of increased effort as helpful and of stressful situations as challenging instead of threatening (e.g., Struthers et al., 2000; Leeming, 2002; Cassady and Gridley, 2005; DeVaney, 2010; McDaniel et al., 2011; Jamieson et al., 2016; Khng, 2017; see also Table 1 in Appendix B for potential starting points regarding linkages among participants evaluations of learning situations and their stress perceptions). Future work could also explore how long-lasting and robust the negative effects of tests on stress perceptions are.

Finally, we would also like to point out that—because our study was conducted slightly before the COVID-19-outbreak and the resulting restriction and thereby triggered changes concerning students daily lives and their learning experiences—findings of replications and future studies might differ due to these interim events: For instance, recent work showed that students had to adjust to remote learning in response to the pandemic and that as a result their achievement goals, engagement, and perceptions of academic success decreased during his time (e.g., Daniels et al., 2021). Orlov et al. (2021) similarly described that students performed, on average, worse during the pandemic than during previous semesters. Concerning students stress perceptions, the results are not that clear: Whereas some studies found that stress and anxiety perceptions generally increased (see, e.g., Limcaoco et al., 2020; Wu et al., 2021; Yang et al., 2021), some work showed that academic stress first increased but then decreased to pre-COVID levels (see, e.g., Charles et al., 2021). Other studies even yielded that studying during COVID-19 had no effects on students’ stress perceptions triggered by learning processes (see, e.g., de la Fuente et al., 2021). Zhang and Liu (2021) further showed that students attitudes toward digital learning influenced the levels of distress they experienced due to the COVID-19 pandemic. Hence, although the findings are not consistent, they highlight that it would be valuable to explore if students stress perceptions or experiences and evaluations of tests (especially regarding remote or digital learning tests) changed in the interim and if these changes might impact their effectiveness. Thus, focusing more strongly on e-learning—as the momentarily most prominent form of learning—seems to be extremely relevant. In line with this, the COVID-19 pandemic and the resulting transfer to remote e-learning also illustrated, among others, the importance and general need for more computerized learning strategies, for more technological applications or digital technologies while learning, or for more innovative, interactive, and gamified teaching strategies to successfully adapt to the current situation and to successfully move to online teaching (see, e.g., Adedoyin and Soykan, 2020; Fergus, 2020; König et al., 2020; Sarju, 2020; Muthuprasad et al., 2021; Nieto-Escamez and Roldán-Tapia, 2021; Obrero-Gaitán et al., 2021; Pozo et al., 2021; Yu et al., 2021). Future work could accordingly investigate the effects of new technologies and of digital learning on education in general but specifically on the application of normally beneficial tests. Hence, future work might focus on, among others, computerized learning and testing, automated scoring systems for tests, automated test question generation, the usage of artificial intelligence in learning, AI-based learning assistants, intelligent tutoring systems, or cyber physical systems in general (see, e.g., Park and Choi, 2008; Grimaldi and Karpicke, 2014; Bachir et al., 2019; Matayoshi et al., 2020; Pugh et al., 2020; Schmohl et al., 2020; Nouhan et al., 2021; see also Radanliev et al., 2020, for a literature review of challenges in the application of artificial intelligence in cyber physical systems). It is thus even more important to conduct further work and to obtain more recent data concerning the in this paper identified issues.

### Conclusion

All in all, our work showed that the application of tests as a desirable difficulty improves later learning outcomes compared to re-reading of the same materials. This applies to curricular and complex learning materials as well as to realistic and difficult test questions and was even independent of participants’ intelligence-estimate. However, the application of such beneficial tests also resulted in higher immediate stress perceptions—even though the test was conducted as a short, low-stakes learning situation. This indicates that actual learning situations including tests might lead to even higher stress perceptions. These stress perceptions were, in turn, linked to diminished benefits of tests. More specifically, controlling for such stress perceptions showed that (at least in this sample) the applied test was even more beneficial when it was not perceived as stressful—or at least as only averagely stressful. Moreover, although there were no moderating effects, higher intelligence was again linked to higher learning outcomes and was even associated with lower immediate stress perceptions during the learning situation.

Hence, our work highlighted important research issues and resulted in interesting findings. Nonetheless, future work is still needed to replicate our study with a much bigger and more diverse sample to explore the robustness of the found effects, to generalize our findings, and to be able to give empirically well-grounded recommendations to lecturers. Moreover, future research should take a closer look at potentially moderating effects of intelligence to ascertain if these effects exist or not. Future work could also try to reduce stress perceptions caused by tests.

## Data Availability Statement

The raw data supporting the conclusions of this article will be made available by the authors, without undue reservation.

## Ethics Statement

Ethical review and approval was not required for the study on human participants in accordance with the local legislation and institutional requirements. The participants provided their written informed consent to participate in this study.

## Author Contributions

KW and M-AR contributed to the study conception and design. Material preparation, data collection, and analyses were performed by KW. Funding acquisition and supervision was performed by M-AR. The first draft of the manuscript was written by KW and M-AR, and KW and M-AR commented on previous versions of the manuscript. All authors read and approved the final manuscript.

## Conflict of Interest

The authors declare that the research was conducted in the absence of any commercial or financial relationships that could be construed as a potential conflict of interest.

## Publisher’s Note

All claims expressed in this article are solely those of the authors and do not necessarily represent those of their affiliated organizations, or those of the publisher, the editors and the reviewers. Any product that may be evaluated in this article, or claim that may be made by its manufacturer, is not guaranteed or endorsed by the publisher.

## Footnotes

^1^Our study was pre-registered by AsPredicted (see ).

^2^The procedure of the mini-q was—in accordance with Prof. Dr. Tanja Baudson—slightly adapted: Instead of letting participants solve all tasks without a time limit and to then use the number of correctly solved tasks at exactly 3 min, we directly terminated the measurement after 3 min.

^3^Exploratively conducted (hierarchical regression) analyses further supported—at least concerning this study and this sample—the assumed linear (instead of a polynominal/non-linear) relation between participants stress perceptions and their later learning outcomes: Neither a regression model assuming a quadratic nor a regression model assuming a cubic link between stress and later learning outcomes was able to explain more variance than a model assuming a linear relation [both *ps* ≥ 0.342; see also Sotardi et al., 2020 regarding this approach and similar findings].

^4^In line with typically used wordings regarding mediation analyses, we will also refer to the regression analysis testing the effect of the predictor (learning condition) on the potential mediator (stress perceptions) as *path a* and to the regression analysis testing the effect of the mediator (stress perceptions) on the criterion (learning outcomes) as *path b*. We also refer to the effect of the predictor (learning condition) without controlling for the mediator (stress perceptions) on the criterion (learning outcomes) as *path c* (*total effect*) and to the effect of the predictor (learning condition) on the criterion (learning outcomes) while controlling for the potential mediator (stress perceptions) as *path c’* (*direct effect*). We also refer to the indirect effect of the predictor (learning condition) on the criterion (learning outcomes) *via* the mediator (stress perceptions) as *path a x path b*.

^5^We would like to thank an anonymous reviewer for this suggestion.

^6^Interestingly, intelligence was also positively correlated with participants number of correctly answered test questions in the test condition (*N* = 42, *r* = 0.40, *p* = 0.008). The number of correctly answered test questions was then, in turn, positively correlated with participants long-term learning (*N* = 42, *r* = 0.86, *p* < 0.001) and negatively with their acute stress perceptions (*N* = 42, *r* = −0.54, *p* < 0.001). These findings highlight the importance of students’ successfulness while working on difficult learning tests and the importance of their (cognitive) abilities to solve such difficult tasks (see also Richland et al., 2005).

## Appendix a – Materials

Materials and example items (translated for this presentation, used materials in German)

Example questions of the questions applied in the learning test in the test condition:

1. What is meant by cerebral dominance?

*Please answer the question in one or two sentences at most*.

2. In apraxia, which type of motor function/movement is disturbed: __________________

3. What should the patient enumerate during the sodium amytal test?

(a) Nothing

(b) Difficult things (e.g., answers to complex math problems, statements,…)

(c) Well-known things (e.g., the letters of the alphabet, the days of the week,…)

(d) Made-up things (e.g., freely invented names,…)

Manipulation check questions applied at the end of Session 1:

1. How difficult did you find working on the second learning phase? one (*very easy*) to five (*very difficult*).

2. How helpful for retaining the learning material did you find working on the second learning phase? one (*not helpful at all*) to five (*very helpful*).

3. How (cognitively) strenuous did you find working on the second learning phase? one (*not strenuous at all*) to five (*very strenuous*).

4. How would you most likely evaluate the second learning phase? As …, one (*a challenge*) to five (*a threat*).

5. How would you best describe the second learning phase? As…, one (*extremely negative*) to five (*extremely positive*).

6. How well do you think you have worked through the second learning phase? one (*very poor*) to five (*very well*).

## Appendix B – Exploratory Analyses

Exploratory analyses focusing on the three different types of final test questions:

Considering only the final test questions that were identical to the questions posed in the learning test in Session 1 (following: *identical final test questions*), participants were on average able to give 6.51 of 11 (59.18%) correct answers (*SD*=2.08, range: 1–10). We then conducted a *t*-test to compare later learning outcomes indicated only by the identical final test questions for participants in both learning conditions: *M_re-reading_*=6.00, *SD_re-reading_*=2.16, *M_test_*=7.07, *SD_test_*=1.87, *t*(87)=−2.49, *p*=0.015, *d*=−0.53 (95% CI[−0.95; −0.10]). As assumed, participants in the test condition answered more identical final test questions correctly than participants in the re-reading control condition. The size of this effect can be interpreted as medium.

Considering only the final test questions that were slightly changed versions of questions posed in the learning test in Session 1 to assess transfer (following: *transfer final test questions*), participants were on average able to give 3.41 of 9 (37.89%) correct answers (*SD*=1.80, range: 0–8). We then conducted a *t*-test to compare later learning outcomes indicated only by the transfer final test questions for participants in both learning conditions: *M_re-reading_*=3.02, *SD_re-reading_*=1.60, *M_test_*=3.83, *SD_test_*=1.92, *t*(87)=−2.18, *p*=0.032, *d*=−0.46 (95% CI[−0.88; −0.04]). As assumed, participants in the test condition answered more transfer final test questions correctly than participants in the re-reading control condition. The size of this effect can be interpreted as medium.

Considering only the final test questions that were new and focused on information that were presented in the textbook chapter but that had not been implemented in the learning test in Session 1 (following: *new final test questions*), participants were on average able to give 3.93 of 7 (56.14%) correct answers (*SD*=4.43, range: 1–7). We then conducted a *t*-test to compare later learning outcomes indicated only by the new final test questions for participants in both learning conditions: *M_re-reading_*=3.85, *SD_re-reading_*=1.33, *M_test_*=4.03, *SD_test_*=1.54, *t*(87)=−0.57, *p*=0.572, *d*=−0.12 (95% CI[−0.54; 0.27]). Participants in the test condition did not significantly answer more new final test questions correctly than participants in the re-reading control condition.

Notably, these explorative findings indicate that the beneficial effects of tests only arise for information that were actually worked on during the learning test and not for information that participants read in the initial study opportunity but that had not been part of the learning test.

Exploratory correlational analyses showed that participants stress perceptions were negatively correlated to identical final test questions (*r*=−0.18, *p*=0.095), transfer final test questions (*r*=−0.26, *p=0.*014; showing a small to medium correlation), and new final test questions (*r*=−0.20, *p=0*.055). Notably, only the correlation of transfer final test questions and participants stress perception reached significance when using two-sided tests (the correlations among stress perceptions and identical as well as new final test questions can be described as marginally significant and reached significance when using one-sided tests). Further exploratory analyses yielded that the three correlation coefficients did not significantly differ from each other (all *p*s≥0.232).

Further exploratory correlational analyses found that participants’ intelligence-estimates were significantly and positively correlated to identical final test questions (*r*=0.28, *p*=0.009), transfer final test questions (*r*=0.30, *p=0.*005), and new final test questions (*r*=0.25, *p=0*.017). Thus, higher intelligence-estimates were generally linked to higher later learning outcomes for the three different types of final test questions (showing medium correlations). Further exploratory analyses showed that the three correlation coefficients did not significantly differ from each other (all *p*s≥0.321).

Exploratory analyses focusing on the correlations among participant stress perceptions and the six questions checking the manipulation of the two learning conditions:

**Table 1:** Exploratory correlations among the six manipulation check questions and participants stress perceptions (*N*=89).

	1	2	3	4	5	6	7
1. Difficulty	—						
2. Helpfulness	−0.17	—					
3. Strenuousness	0.59^**^	0.14	—				
4. Evaluation – challenge/threat	−0.13	−0.21^*^	−0.18	—			
5. Evaluation – negative/positive	−0.43^**^	0.60^**^	−0.11	−0.05	—		
6. Successfulness	−0.66^**^	0.35^**^	−0.36^**^	0.09	0.51^**^	—	
7. Stress perceptions	0.51^**^	−0.28^**^	0.31^**^	0.04	−0.44^**^	−0.67^**^	—

* *p< 0.05*;

** *p< 0.01*

See Appendix A for a full list of the manipulation check questions.
